# Evaluating the role of insulin resistance in chronic intestinal health issues: NHANES study findings

**DOI:** 10.3389/fnut.2025.1602922

**Published:** 2025-05-26

**Authors:** Dongyao Zhao, Meihua Zhao, Bing Gao, He Lu

**Affiliations:** ^1^Department of Gastroenterology, The First Affiliated Hospital of Zhengzhou University, Zhengzhou, China; ^2^Department of Gastroenterology, The Second Affiliated Hospital of Zhengzhou University, Zhengzhou, China; ^3^Department of Cardiology, The People’s Hospital of Jiawang District of Xuzhou, Xuzhou, China

**Keywords:** intestinal health, chronic diarrhea, chronic constipation, insulin resistance surrogate indices, NHANES (National Health and Nutrition Examination Survey)

## Abstract

**Background:**

Intestinal health issues affect approximately 20% of the global population, yet the relationship between insulin resistance (IR) and intestinal health remains poorly understood. This study evaluated the discriminative ability of five IR surrogate indices—homeostatic model assessment for insulin resistance (HOMA-IR), triglyceride-glucose index (TyG), TyG adjusted for body mass index (TyG-BMI), triglyceride-to-high-density lipoprotein cholesterol ratio (TG/HDL-C), and estimated glucose disposal rate (eGDR)—for chronic diarrhea and constipation in adults.

**Methods:**

Using data from the 2005–2010 National Health and Nutrition Examination Survey (NHANES), we analyzed associations between five IR surrogate indices and chronic diarrhea/constipation in adults. Key variables were selected via the Boruta algorithm and incorporated into weighted multivariate logistic regression models. Restricted cubic spline (RCS) analysis, threshold effect analysis, and receiver operating characteristic (ROC) curves were employed to assess these associations.

**Results:**

Among 6,133 participants in this study, 7.5% had chronic diarrhea and 7.4% had chronic constipation. After adjusting for confounders, multivariate logistic regression revealed significant positive associations of HOMA-IR (OR: 1.02, 95% CI: 1.00–1.04), TyG (OR: 1.28, 95% CI: 1.05–1.55), and TyG-BMI (OR: 1.01, 95% CI: 1.00–1.01) with chronic diarrhea, while eGDR showed an inverse association (OR: 0.88, 95% CI: 0.80–0.96). No significant associations were observed between IR surrogate indices and chronic constipation. RCS and threshold effect analyses demonstrated a non-linear relationship between TG/HDL-C and chronic diarrhea: Each 1-unit increase in TG/HDL-C below the threshold of 7.33 elevated diarrhea risk by 11% (95% CI: 1.05–1.17). ROC analysis indicated that TyG-BMI (AUC: 0.656 vs. 0.644) and eGDR (AUC: 0.652 vs. 0.644) significantly improved the discriminative ability of the baseline model for chronic diarrhea, whereas HOMA-IR and TyG showed no statistically meaningful enhancements.

**Conclusion:**

IR surrogate indices were significantly associated with chronic diarrhea but not chronic constipation, highlighting their potential as biomarkers for screening diarrhea in the general population.

## 1 Introduction

In recent years, there has been an increasing recognition of the importance of intestinal health, particularly regarding the prevalent issues of chronic diarrhea and constipation. Approximately 20% of adults are diagnosed with chronic diarrhea, while approximately 17% experience constipation in the general population (1, 2). These health challenges significantly impact individuals’ quality of life and contribute to both direct and indirect costs associated with treatment and reduced work productivity (3, 4). Individuals suffering from chronic diarrhea and constipation face heightened risks of metabolic and cardiovascular conditions and are also more likely to develop colorectal cancer and experience all-cause mortality (5, 6). Despite the serious implications of these disorders, timely interventions and effective management strategies are often neglected in clinical practice. Therefore, it is imperative to elevate awareness within primary care regarding the burden of these diseases and to advocate for early diagnosis, facilitating the formulation of targeted prevention and control measures.

The mechanisms behind chronic diarrhea and constipation are still not fully understood. Earlier research has revealed various factors that contribute to the development of chronic diarrhea and constipation, such as poor dietary habits (7), bacterial infections (8), intestinal dysbiosis (9, 10), and psychological issues such as anxiety and depression (9, 11). A growing amount of evidence shows that abnormal intestinal habits are closely linked to metabolic disorders. People who have diabetes (12), non-alcoholic fatty liver disease (5), metabolic syndrome (6), and obesity (13) are more vulnerable to experiencing chronic diarrhea and constipation. A shared pathological mechanism connecting these conditions is insulin resistance (IR). Lipopolysaccharides (LPSs) that originate from gut microbiota can trigger IR through the activation of Toll-like receptor 4 (TLR4) (14). In addition, substantial evidence supports a causal relationship between chronic diarrhea, constipation, and intestinal dysbiosis (9, 10). As a result, this study speculates an association between IR and the prevalence of chronic diarrhea and constipation.

The hyperinsulinemic-euglycemic clamp technique is widely recognized as the gold standard for assessing IR; however, its complex procedures and high costs significantly limit its widespread clinical application (15). Therefore, developing cost-effective and easily accessible surrogate markers for IR is of great practical significance. In recent years, several IR surrogate indices have been introduced into clinical practice, including homeostatic model assessment for insulin resistance (HOMA-IR), triglyceride-glucose index (TyG), TyG adjusted for body mass index (TyG-BMI), triglyceride-to-high-density lipoprotein cholesterol ratio (TG/HDL-C), and estimated glucose disposal rate (eGDR). These indices each have unique advantages: TyG and TyG-BMI excel in evaluating lipid metabolism-related IR, eGDR comprehensively reflects glucose disposal capacity, and TG/HDL-C integrates multiple metabolic parameters to provide a more comprehensive assessment (16–20). Nevertheless, existing research has primarily focused on the relationship between IR and chronic metabolic diseases such as diabetes, obesity, and cardiovascular diseases, while studies exploring the association between IR and intestinal health remain limited. This study was the first to systematically evaluate the correlation between these five IR surrogate indices and chronic diarrhea and constipation, aiming to identify novel biomarkers for early diagnosis and intervention of these conditions, thereby addressing a critical gap in the field of IR surrogates and intestinal health.

## 2 Methods

### 2.1 Study population

**Figure 1:**
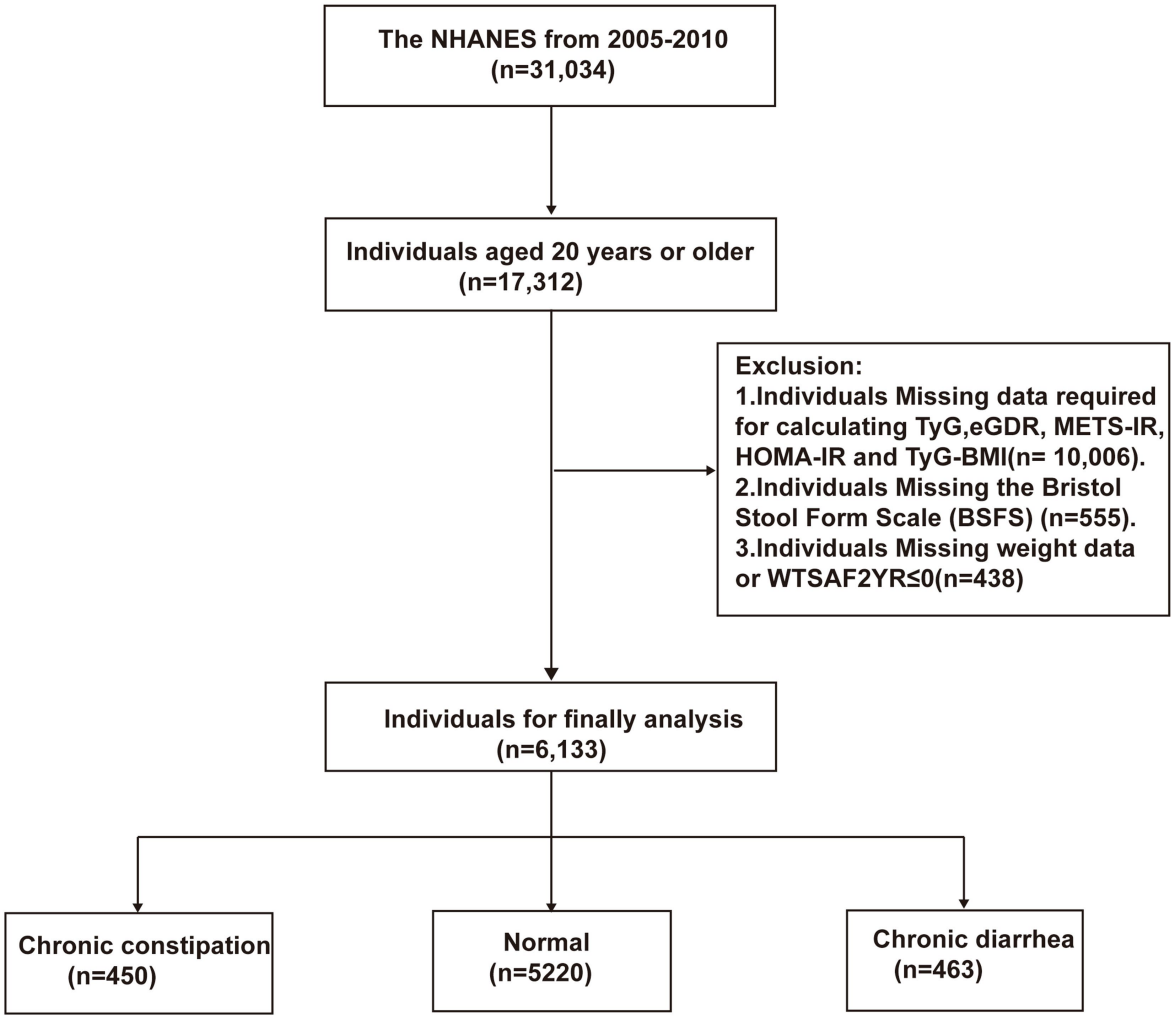
Flow diagram of patient selection.

This study included 31,034 participants from the National Health and Nutrition Examination Survey (NHANES) conducted between 2005 and 2010. NHANES, approved by the Ethics Review Board of the National Center for Health Statistics, is designed to systematically investigate the nutritional and health status of U.S. citizens biennially. After providing informed consent, participants completed the questionnaires, physical examinations, and biological sample collection, with the assistance of trained technicians. Final examination reports were reviewed by staff, anonymized to protect participant privacy, and publicly released on the NHANES website. Figure 1 outlines the participant selection procedure for this research. Exclusion criteria included (1) individuals <20 years old (*n* = 13,722); (2) those lacking essential data for five IR surrogate indices calculations (*n* = 10,006); (3) individuals with missing Bristol Stool Form Scale (BSFS) data (*n* = 555); and (4) individuals with missing weight data or WTSAF2YR ≤ 0 (*n* = 618).

### 2.2 IR surrogate indices

This study incorporated five IR surrogate indices; the detailed calculation formulas for these indices are presented in Table 1 (16–20). Published studies have recommended stratifying eGDR using specific cutoff values (<4, 4–6, 6–8, and ≥8 mg/kg/min) (21, 22) as these categories have been shown to reflect significant differences in mortality rates among individuals with diabetes. Conversely, due to the lack of established diagnostic thresholds for the remaining four IR surrogate indices, these parameters were categorized via quartile-based stratification.

**Table 1:** Calculation formula of the five novel IR surrogate indices.

IR surrogate indices	Calculation formula
HOMA-IR	HOMA-IR = (Fasting insulin (μU/mL) × FBG (mg/dL))/405 (18)
TyG	TyG = ln (0.5*TG (mg/dL) × FBG (mg/dL)) (18)
TyG-BMI	TyG-BMI = TyG × BMI (kg/m^2^) (19)
TG/HDL	TG (mg/dL)/ HDL (mg/dL) (20)
eGDR	eGDR = 19.02 − (0.22 × BMI (kg/m^2^)) − (3.26 × Hypertension) − (0.61 × HbA1c (%)) (Hypertension: yes 1; No 0) (16)

### 2.3 Assessment of chronic diarrhea and chronic constipation

In this study, chronic diarrhea and constipation were assessed using BSFS (Table 2) (23). Participants were classified as having chronic diarrhea if their usual or most common stool type was categorized as Type 6–7, or as having chronic constipation if their stool type was categorized as Type 1–2. All other stool types were considered normal.

**Table 2:** Assessment of chronic constipation and chronic diarrhea based on BSFS.

Chronic intestinal disease	BSFS
Chronic constipation	Type 1 (separate hard lumps, like nuts) Type 2 (sausage-like, but lumpy)
Normal	Type 3 (like a sausage but with cracks in the surface) Type 4 (like a sausage or snake, smooth and soft) Type 5 (soft blobs with clear-cut edges)
Chronic diarrhea	Type 6 (fluffy pieces with ragged edges, a mushy stool) Type 7 (watery, no solid pieces)

### 2.4 Covariates

The covariates in this study included general participant information such as age, gender, race/ethnicity, education level, marital status (married or living with a partner, and unmarried or separated), and the poverty-to-income ratio (PIR) (<1.3, 1.3–3.5, ≥3.5) (24). BMI was calculated as weight in kilograms divided by height in meters squared (kg/m^2^) (25). Laboratory measurements included serum fasting blood glucose (FBG), which was assessed using the Roche/Hitachi Cobas C 501 Chemistry Analyzer (Roche Diagnostics, Indianapolis, IN, United States) before 2015, employing a hexokinase-mediated enzymatic reaction. In 2015, the laboratory instrumentation was updated, with the Roche C501 replaced by the Roche C311 Chemistry Analyzer (Roche Diagnostics, Indianapolis, IN, United States). A correction equation was applied to adjust glucose measurements between the two instruments, following official guidelines. Glycated hemoglobin (HbA1c) was measured using the Tosoh G8 Glycohemoglobin Analyzer (HLC-723G8), based on high-performance liquid chromatography (HPLC). Serum insulin concentrations were determined using the Tosoh AIA-900 Automated Immunoassay Analyzer (Tosoh Bioscience, Tokyo, Japan), employing an immunoenzymometric assay (IEMA). Liver function tests were conducted using the DxC800 system, a fully automated clinical chemistry analyzer developed by Beckman Coulter. High-density lipoprotein cholesterol (HDL-C), low-density lipoprotein cholesterol (LDL-C), triglycerides (TGs), and total cholesterol (TC) were measured using the Roche Cobas 6,000 Chemistry Analyzer (Roche Diagnostics, Indianapolis, IN, United States). Behavioral habits and comorbidities were assessed as follows: Smoking behavior was classified according to participants’ lifetime smoking patterns: non-smoker (fewer than 100 cigarettes ever smoked), former smoker (over 100 cigarettes smoked but abstinent for at least 1 year), and current smoker (over 100 cigarettes smoked and actively smoking) (26). Alcohol consumption was defined as having at least 12 drinks of any type of alcoholic beverage in the past year, where one drink was equivalent to 12 oz. of beer, 5 oz. of wine, or 1.5 oz. of liquor (24). Physical activity (PA) was categorized into four levels based on metabolic equivalent of task (MET) metrics: (1) no PA (0 MET·min/week); (2) low PA (<600 MET·min/week); (3) moderate PA (600–1,200 MET·min/week); and (4) high PA (>1,200 MET·min/week) (27).

Participants’ mental health status was determined using the Patient Health Questionnaire-9 (PHQ-9). PHQ-9 score ≥10 was considered indicative of depressive symptoms, while scores below 10 were classified as normal (28). Diabetes was defined as meeting one or more of the following criteria: (1) a previous diagnosis of diabetes by a healthcare professional or current use of insulin or other glucose-lowering medications; (2) FBG ≥ 7 mmol/L; and/or (3) HbA1c ≥ 6.5% (29). The definition of hypertension encompassed fulfillment of either diagnostic parameter: (1) self-reported physician-diagnosed hypertension or active pharmacological management with antihypertensive agents; (2) and sustained blood pressure elevation evidenced by triplicate measurements demonstrating systolic pressure ≥140 mmHg and/or diastolic values ≥90 mmHg, calculated through averaged readings (30).

### 2.5 Statistical analysis

Considering the complex, multi-stage sampling design of NHANES, sample weights were incorporated into the subsequent statistical analyses. Based on NHANES recommendations, the fasting subsample weight (WTSAF2YR) divided by the number of cycles was applied as the sample weight for this study (24). During data processing, participants with missing key variables were excluded, and the specific selection process is illustrated in Figure 1. For participants missing other covariates, multiple imputation using the random forest method was performed. The missing covariates and their proportions are detailed in Supplementary Table 1. Continuous variables were presented as mean ± standard deviation (SD), while categorical variables were expressed as proportions. The analysis of one-way ANOVA (for continuous variables) or chi-square tests (for categorical variables) was used to compare clinical data of participants grouped by intestinal health status. The Boruta algorithm, a supervised machine learning method based on random forests, was employed to identify covariates genuinely associated with intestinal health for inclusion in subsequent multivariate logistic regression models. Notably, to avoid the impact of multicollinearity, test indicators used to calculate independent variables were not individually included in the model. Furthermore, multicollinearity was assessed, and covariates with a variance inflation factor (VIF) > 5 were sequentially excluded to eliminate multicollinearity with independent variables. Multivariate logistic regression analysis was used to examine the associations between the five IR surrogate indices (analyzed as both continuous and categorical variables) and chronic diarrhea or chronic constipation. Model 1 was unadjusted for any variables. Model 2 was adjusted for age, gender, race/ethnicity, education level, marital status, and PIR. Model 3 was further adjusted for LDL-C, smoking status, alcohol consumption, diabetes, hypertension, mental health status, and PA based on Model 2.

Four-knot restricted cubic spline (RCS) plots were used to capture the dose–response relationships between the five IR surrogate indices and chronic diarrhea or constipation. If non-linear relationships were observed, threshold effect analysis was further conducted to examine the associations on either side of the inflection point. Receiver operating characteristic (ROC) curves were used to evaluate whether HOMA-IR, TyG, TyG-BMI, TG/HDL, and eGDR could improve the predictive ability of the baseline risk model for chronic diarrhea or chronic constipation. This study utilizes version R.4.3.0 for data analysis, with a two-tailed *p*-value less than 0.05 considered statistically significant.

## 3 Results

### 3.1 Clinical characteristics

A total of 6,133 participants were enrolled in this study, of whom 7.5% (*n* = 463) had chronic diarrhea and 7.4% (*n* = 450) had chronic constipation. The clinical characteristics of the participants, stratified by intestinal health status, are summarized in Table 3. The mean age of the participants was 49.37 ± 17.82 years, with 49.2% being male. Compared to the normal group, the chronic diarrhea group showed a lower proportion of male participants (44.1% vs. 51.4%), Mexican Americans (23.3% vs. 18.1%), individuals with less than a high school education (41.0% vs. 25.7%), lower income levels (37.4% vs. 28.2%), alcohol consumption (24.8% vs. 15.7%), and physical inactivity (36.9% vs. 29.3%). In addition, the chronic diarrhea group had a higher prevalence of hypertension (57.5% vs. 45.9%), diabetes (24.8% vs. 15.7%), and depressive symptoms (16.6% vs. 7%). Notably, the chronic diarrhea group demonstrated significantly elevated levels of HOMA-IR (4.57 ± 5.19 vs. 3.52 ± 4.33), TyG (8.81 ± 0.68 vs. 8.63 ± 0.67), TyG-BMI (269.82 ± 70.26 vs. 250.50 ± 64.06), and TG/HDL (3.25 ± 2.90 vs. 2.99 ± 5.14), alongside reduced eGDR levels (6.71 ± 2.77 vs. 7.55 ± 2.63), compared to the normal group (*p* < 0.001).

**Table 3:** Clinical characteristics of participants based on intestinal health.

Characteristic		Chronic intestinal disease			
	Total	None	Chronic constipation	Chronic diarrhea	*P-*value
*N* (weighted)	192,309,303	166,256,247	13,168,032	12,885,024	
*n* (un-weighted)	6,133	5,220 (85.1)	450 (7.4)	463 (7.5)	
Male (%)	3,016 (49.2)	2,685 (51.4)	127 (28.2)	204 (44.1)	<0.001
Race (%)					<0.001
Mexican American	1,146 (18.7)	943 (18.1)	95 (21.1)	108 (23.3)	
Other Hispanic	555 (9.0)	454 (8.7)	50 (11.1)	51 (11.0)	
Non-Hispanic White	3,059 (49.9)	2,660 (51.0)	196 (43.6)	203 (43.8)	
Non-Hispanic Black	1,124 (18.3)	947 (18.1)	92 (20.4)	85 (18.4)	
Other/multiracial	249 (4.1)	216 (4.1)	17 (3.8)	16 (3.5)	
Education (%)					<0.001
Less than high school	1,673 (27.3)	1,342 (25.7)	141 (31.3)	190 (41.0)	
High school graduate	1,485 (24.2)	1,257 (24.1)	125 (27.8)	103 (22.2)	
Some college or above	2,975 (48.5)	2,621 (50.2)	184 (40.9)	170 (36.7)	
PIR					<0.001
<1.3	1,808 (29.5)	1,471 (28.2)	164 (36.4)	173 (37.4)	
1.3–3.5	2,370 (38.6)	2,005 (38.4)	185 (41.1)	180 (38.9)	
≥3.5	1,955 (31.9)	1,744 (33.4)	101 (22.4)	110 (23.8)	
Marry status					<0.001
Married/living with others	2,273 (37.1)	1,923 (36.8)	182 (40.4)	168 (36.3)	
Unmarried/separated	3,860 (62.9)	3,297 (63.2)	268 (59.6)	295 (63.7)	
Drinking (%)	998 (16.3)	819 (15.7)	64 (14.2)	115 (24.8)	<0.001
Smoke (%)					<0.001
No smoker	3,246 (52.9)	2,732 (52.3)	287 (63.8)	227 (49.0)	
Former smoker	1,283 (20.9)	1,099 (21.1)	71 (15.8)	113 (24.4)	
Current smoker	1,604 (26.2)	1,389 (26.6)	92 (20.4)	123 (26.6)	
Hypertension (%)	2,856 (46.6)	2,398 (45.9)	192 (42.7)	266 (57.5)	<0.001
Diabetes (%)	998 (16.3)	819 (15.7)	64 (14.2)	115 (24.8)	<0.001
PA					<0.001
No-PA	1,858 (30.3)	1,529 (29.3)	158 (35.1)	171 (36.9)	
LLPA	1,413 (23.0)	1,216 (23.3)	102 (22.7)	95 (20.5)	
MLPA	786 (12.8)	681 (13.0)	52 (11.6)	53 (11.4)	
HLPA	2,076 (33.8)	1,794 (34.4)	138 (30.7)	144 (31.1)	
Depression (%)	492 (8.0)	364 (7.0)	51 (11.3)	77 (16.6)	<0.001
Age (years)	49.37 (17.82)	49.33 (17.82)	46.86 (19.06)	52.27 (16.24)	<0.001
BMI (kg/m2)	28.97 (6.53)	28.89 (6.46)	28.27 (6.64)	30.48 (7.00)	<0.001
FBG (mg/dl)	107.53 (33.72)	107.27 (33.00)	104.36 (36.78)	113.55 (37.76)	<0.001
HbA1c	5.69 (1.04)	5.68 (1.03)	5.63 (1.09)	5.86 (1.15)	<0.001
AST (u/l)	26.13 (17.12)	26.35 (18.00)	24.07 (9.68)	25.61 (11.59)	<0.001
ALT (u/l)	25.92 (18.49)	26.14 (18.92)	22.99 (15.15)	26.17 (16.15)	<0.001
TG (mg/dl)	197.66 (42.64)	197.45 (42.60)	201.20 (44.45)	196.57 (41.31)	<0.001
TC (mg/dl)	132.02 (120.56)	130.74 (124.35)	133.00 (95.56)	145.41 (95.83)	<0.001
HDL-C (mg/dl)	54.27 (16.28)	54.18 (16.29)	57.13 (16.13)	52.51 (15.89)	<0.001
LDL-C (mg/dl)	116.41 (35.85)	116.55 (35.93)	116.11 (35.59)	115.12 (35.21)	<0.001
HOMA-IR	3.59 (4.38)	3.52 (4.33)	3.38 (3.93)	4.57 (5.19)	<0.001
TyG	8.64 (0.67)	8.63 (0.67)	8.63 (0.69)	8.81 (0.68)	<0.001
TyG-BMI	251.57 (64.92)	250.50 (64.06)	245.24 (66.18)	269.82 (70.26)	<0.001
TG/HDL	2.99 (4.87)	2.99 (5.14)	2.71 (2.79)	3.25 (2.90)	<0.001
eGDR	7.51 (2.65)	7.55 (2.63)	7.90 (2.63)	6.71 (2.77)	<0.001

PIR, poverty index ratio; PA, physical activity; LLPA, low-level physical activity; MLPA, moderate-level physical activity; HLPA, high-level physical activity; BMI, body mass index; FBG, fasting blood glucose; HbA1c, glycosylated hemoglobin type A1C; AST, aspartate aminotransferase; ALT, alanine aminotransferase; TGs, triglycerides; TC, total cholesterol; HDL-C, high-density lipoprotein cholesterol; LDL-C, low-density lipoprotein cholesterol; HOMA-IR, Homeostatic Model Assessment for Insulin Resistance; TyG, triglyceride-glucose index; TyG-BMI, triglyceride-glucose index adjusted for body mass index; eGDR, estimated glucose disposal rate.

Similarly, the chronic constipation group exhibited a lower prevalence of male participants (28.2% vs. 51.4%), Mexican Americans (21.1% vs. 18.1%), individuals with less than a high school education (31.3% vs. 25.7%), lower income levels (36.4% vs. 28.2%), physical inactivity (35.1% vs. 29.3%), and depressive symptoms (11.3% vs. 7%). The chronic constipation group exhibited lower levels of HOMA-IR (3.38 ± 3.93 vs. 3.52 ± 4.33), TyG-BMI (245.24 ± 66.18 vs. 250.50 ± 64.06), and TG/HDL (2.71 ± 2.79 vs. 2.99 ± 5.14), while eGDR (6.71 ± 2.77 vs. 7.55 ± 2.63) levels were higher (*p* < 0.001).

### 3.2 Feature selection

**Figure 2:**
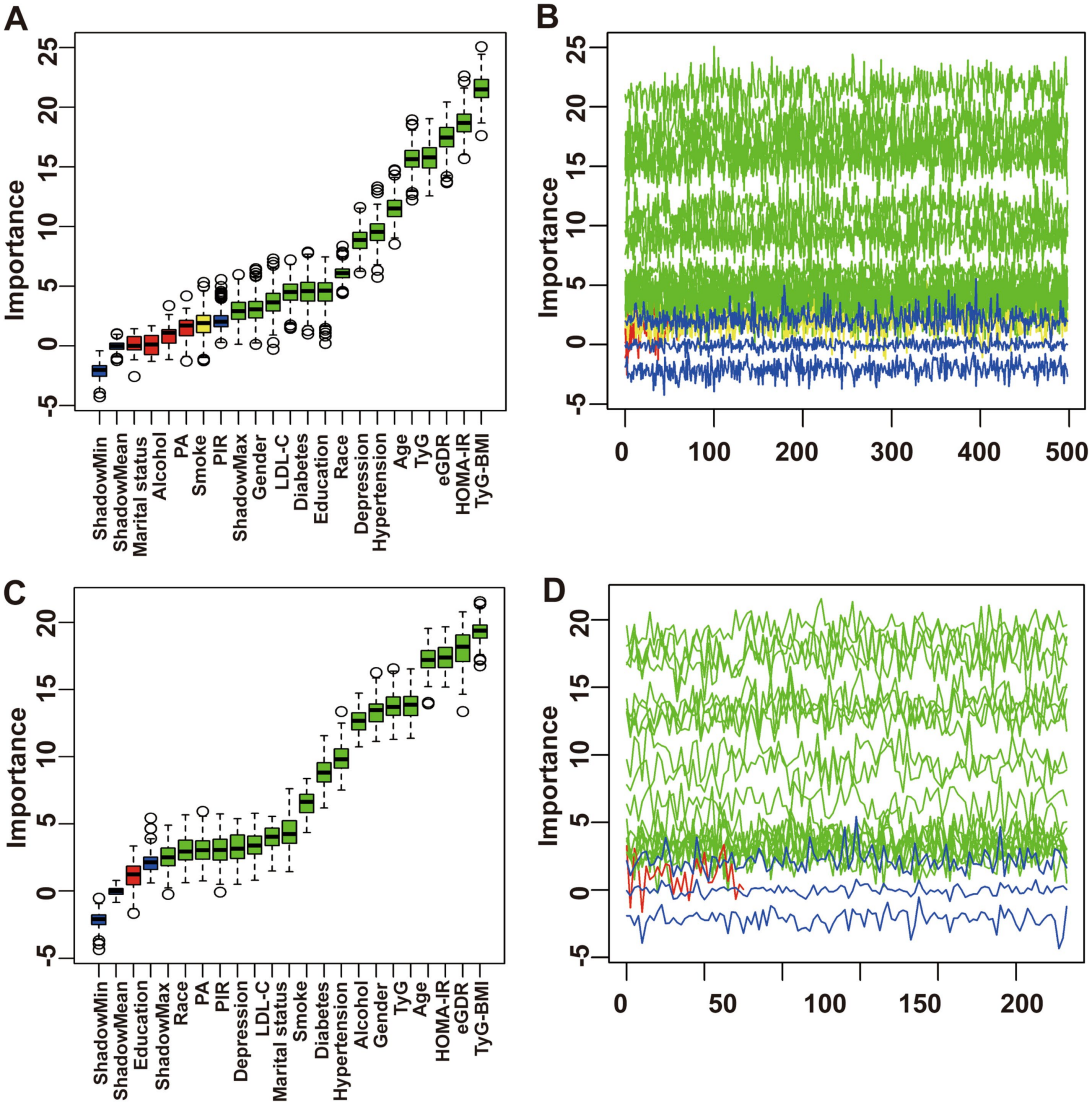
Feature selection process for chronic diarrhea based on Boruta’s algorithm **(A)** and the value evolution of Z-score in the screening process **(B)**. Feature selection process for chronic constipation based on Boruta’s algorithm **(C)** and the value evolution of Z-score in the screening process **(D)**.

The feature selection results based on the Boruta algorithm are presented in Figure 2. After 500 iterations, the top 10 variables most closely associated with chronic diarrhea (ranked by z-score, excluding IR surrogate indices) were identified as age, hypertension, depressive symptoms, race/ethnicity, education level, diabetes, LDL-C, gender, PIR, and smoking status. Similarly, the top 10 parameters most strongly associated with chronic constipation included age, gender, alcohol consumption, hypertension, diabetes, smoking status, marital status, LDL-C, PA, depression and PIR. Based on the feature selection results from the Boruta algorithm and previous research findings, the covariates included in the final logistic regression models were as follows: Model 1 was unadjusted; Model 2 adjusted for age, gender, race/ethnicity, education level, marital status, and PIR; Model 3 further adjusted for LDL-C, smoking status, alcohol consumption, diabetes, hypertension, depressive symptoms, and PA based on Model 2.

### 3.3 Relationship between the IR surrogate indices and chronic diarrhea or chronic constipation

Three logistic regression models were constructed to examine the independent associations between five IR surrogate indices and the risks of chronic diarrhea and constipation (Tables 4, 5). When analyzed as continuous variables, each 1-unit increase in HOMA-IR, TyG, and TyG-BMI was associated with a 2% (95% CI: 1.00–1.04), 28% (95% CI: 1.05–1.55), and 1% (95% CI: 1.00–1.01) increased risk of chronic diarrhea, respectively, after full adjustment for covariates. Conversely, each 1-unit increase in eGDR was associated with a 12% reduction in the risk of chronic diarrhea (95% CI: 0.80–0.96). Similarly, when analyzed as categorical variables, participants in the highest quartile (Q4) of HOMA-IR, TyG, and TyG-BMI exhibited a 50% (95% CI: 1.04–2.17), 65% (95% CI: 1.13–2.42), and 90% (95% CI: 1.28–2.81) increased risk of chronic diarrhea, respectively, compared to those in the lowest quartile (Q1). In contrast, participants in the highest quartile (Q4) of eGDR showed a 44% reduction in the risk of chronic diarrhea (95% CI: 1.13–2.42). However, no significant associations were observed between HOMA-IR, TyG, TyG-BMI, TG/HDL, and eGDR and the risk of chronic constipation.

**Table 4:** Association between IR surrogate indices and chronic diarrhea.

Characteristic	Model 1		Model 2		Model 3	
	OR (95%CI)	*P*-value	OR (95%CI)	*P*-value	OR (95%CI)	*P*-value
TyG	1.44 (1.26, 1.65)	<0.001	1.38 (1.20, 1.60)	<0.001	1.28 (1.05, 1.55)	0.015
Q1	Ref		Ref		Ref	Ref
Q2	1.24 (0.90, 1.73)	0.200	1.21 (0.87, 1.68)	0.300	1.16 (0.82, 1.64)	0.400
Q3	1.30 (0.89, 1.89)	0.200	1.23 (0.83, 1.83)	0.300	1.14 (0.76, 1.71)	0.500
Q4	2.07 (1.55, 2.76)	<0.001	1.90 (1.39, 2.61)	<0.001	1.65 (1.13, 2.42)	0.012
*P* for trend		<0.001		<0.001		0.029
TyG-BMI	1.00 (1.00, 1.01)	<0.001	1.01 (1.00, 1.01)	<0.001	1.01 (1.00, 1.01)	0.003
Q1	Ref		Ref		Ref	Ref
Q2	1.42 (0.99, 2.04)	0.054	1.41 (0.99, 2.01)	0.059	1.42 (0.96, 2.11)	0.075
Q3	1.40 (0.99, 1.98)	0.056	1.34 (0.96, 1.87)	0.088	1.31 (0.90, 1.91)	0.200
Q4	2.24 (1.58, 3.16)	<0.001	2.07 (1.48, 2.91)	<0.001	1.90 (1.28, 2.81)	0.003
*P* for trend		<0.001		0.004		0.019
HOMA-IR	1.04 (1.02, 1.06)	<0.001	1.03 (1.01, 1.06)	0.004	1.02 (1.00, 1.04)	0.032
Q1	Ref		Ref		Ref	Ref
Q2	1.27 (0.88, 1.82)	0.200	1.23 (0.85, 1.78)	0.300	1.23 (0.85, 1.78)	0.300
Q3	1.19 (0.79, 1.80)	0.400	1.14 (0.74, 1.74)	0.500	1.09 (0.70, 1.70)	0.700
Q4	1.9 (1.40, 2.58)	<0.001	1.74 (1.27, 2.38)	0.001	1.50 (1.04, 2.17)	0.031
*P* for trend		<0.001		<0.001		0.072
TG/HDL	1.01 (1.00, 1.02)	0.13	1.00 (0.99, 1.02)	0.400	1.00 (0.98, 1.01)	0.800
Q1	Ref		Ref		Ref	Ref
Q2	1.33 (0.92, 1.92)	0.130	1.3 (0.90, 1.89)	0.200	1.25 (0.84, 1.86)	0.200
Q3	1.25 (0.90, 1.74)	0.200	1.22 (0.87, 1.73)	0.200	1.12 (0.80, 1.57)	0.500
Q4	1.79 (1.33, 2.40)	<0.001	1.75 (1.27, 2.40)	0.001	1.51 (1.04, 2.19)	0.033
*P* for trend		<0.001		<0.001		0.052
eGDR	0.91 (0.87, 0.95)	<0.001	0.91 (0.87, 0.96)	<0.001	0.88 (0.80, 0.96)	0.007
Q1	Ref		Ref		Ref	Ref
Q2	0.73 (0.49, 1.08)	0.110	0.72 (0.48, 1.09)	0.120	0.76 (0.49, 1.19)	0.200
Q3	0.43 (0.27, 0.68)	<0.001	0.44 (0.27, 0.69)	<0.001	0.47 (0.28, 0.79)	0.006
Q4	0.49 (0.35, 0.68)	<0.001	0.54 (0.37, 0.78)	0.002	0.56 (0.27, 1.15)	0.110
*P* for trend		<0.001		0.001		0.038

Model 1: adjust for nothing. Model 2: adjust for age, gender, race, education, marriage status, and PIR. Model 3: further adjust for LDL-C, AST, ALT, smoke, alcohol, diabetes, hypertension, depression, and PA.

**Table 5:** Association between IR surrogate indices and chronic constipation.

Characteristic	Model 1		Model 2		Model 3	
	OR (95%CI)	*P*-value	OR (95%CI)	*P*-value	OR (95%CI)	*P*-value
TyG	0.99 (0.79, 1.24)	>0.90	1.13 (0.89, 1.42)	0.300	1.28 (0.97, 1.70)	0.078
Q1	Ref		Ref		Ref	
Q2	0.99 (0.70, 1.41)	>0.90	1.14 (0.78, 1.65)	0.500	1.19 (0.80, 1.78)	0.400
Q3	0.75 (0.53, 1.05)	0.089	0.90 (0.65, 1.25)	0.500	0.98 (0.67, 1.42)	0.900
Q4	1.07 (0.71, 1.61)	0.800	1.34 (0.86, 2.07)	0.200	1.60 (0.96, 2.69)	0.071
*P* for trend		<0.001		0.366		0.149
TyG-BMI	1.00 (1.00, 1.00)	0.028	1.00 (1.00, 1.00)	0.020	1.00 (1.00, 1.01)	0.055
Q1	Ref		Ref		Ref	
Q2	0.76 (0.57, 1.02)	0.070	0.86 (0.63, 1.18)	0.300	0.86 (0.61, 1.22)	0.400
Q3	0.63 (0.45, 0.88)	0.009	0.70 (0.49, 0.99)	0.046	0.69 (0.47, 1.01)	0.058
Q4	0.61 (0.44, 0.85)	0.004	0.61 (0.44, 0.84)	0.004	0.61 (0.41, 0.92)	0.021
*P* for trend		<0.001		0.003		0.012
HOMA-IR	0.96 (0.92, 1.00)	0.046	0.96 (0.92, 1.00)	0.049	0.96 (0.92, 1.01)	0.100
Q1	Ref		Ref		Ref	
Q2	1.11 (0.79, 1.57)	0.500	1.11 (0.78, 1.59)	0.500	1.08 (0.75, 1.54)	0.700
Q3	0.86 (0.65, 1.14)	0.300	0.90 (0.67, 1.19)	0.400	0.89 (0.65, 1.23)	0.500
Q4	0.80 (0.55, 1.17)	0.200	0.82 (0.56, 1.20)	0.300	0.85 (0.56, 1.30)	0.400
*P* for trend		<0.001		0.189		0.377
TG/HDL-C	0.98 (0.95, 1.02)	0.400	1.00 (0.98, 1.02)	>0.900	1.01 (0.99, 1.02)	0.600
Q1	Ref		Ref		Ref	
Q2	0.90 (0.61, 1.33)	0.600	0.98 (0.67, 1.45)	>0.900	1.01 (0.67, 1.52)	>0.900
Q3	0.83 (0.58, 1.18)	0.300	0.99 (0.69, 1.43)	>0.900	1.06 (0.71, 1.60)	0.800
Q4	0.94 (0.63, 1.40)	0.800	1.22 (0.80, 1.85)	0.400	1.36 (0.84, 2.22)	0.200
*P* for trend		<0.001		0.419		0.239
eGDR	1.05 (1.00, 1.11)	0.041	1.04 (0.99, 1.10)	0.140	1.09 (0.98, 1.21)	0.100
Q1	Ref		Ref		Ref	
Q2	0.72 (0.41, 1.27)	0.300	0.70 (0.39, 1.27)	0.200	0.67 (0.36, 1.25)	0.200
Q3	1.24 (0.68, 2.25)	0.500	1.12 (0.61, 2.05)	0.700	1.08 (0.54, 2.17)	0.800
Q4	1.10 (0.65, 1.86)	0.700	1.04 (0.60, 1.81)	0.900	1.10 (0.49, 2.46)	0.800
*P* for trend		<0.001		0.342		0.397

Model 1: adjust for nothing. Model 2: adjust for age, gender, race, education, marriage status, and PIR. Model 3: further adjust for LDL-C, AST, ALT, smoke, alcohol, diabetes, hypertension, depression, and PA.

### 3.4 The detection of non-linear relationships

**Figure 3:**
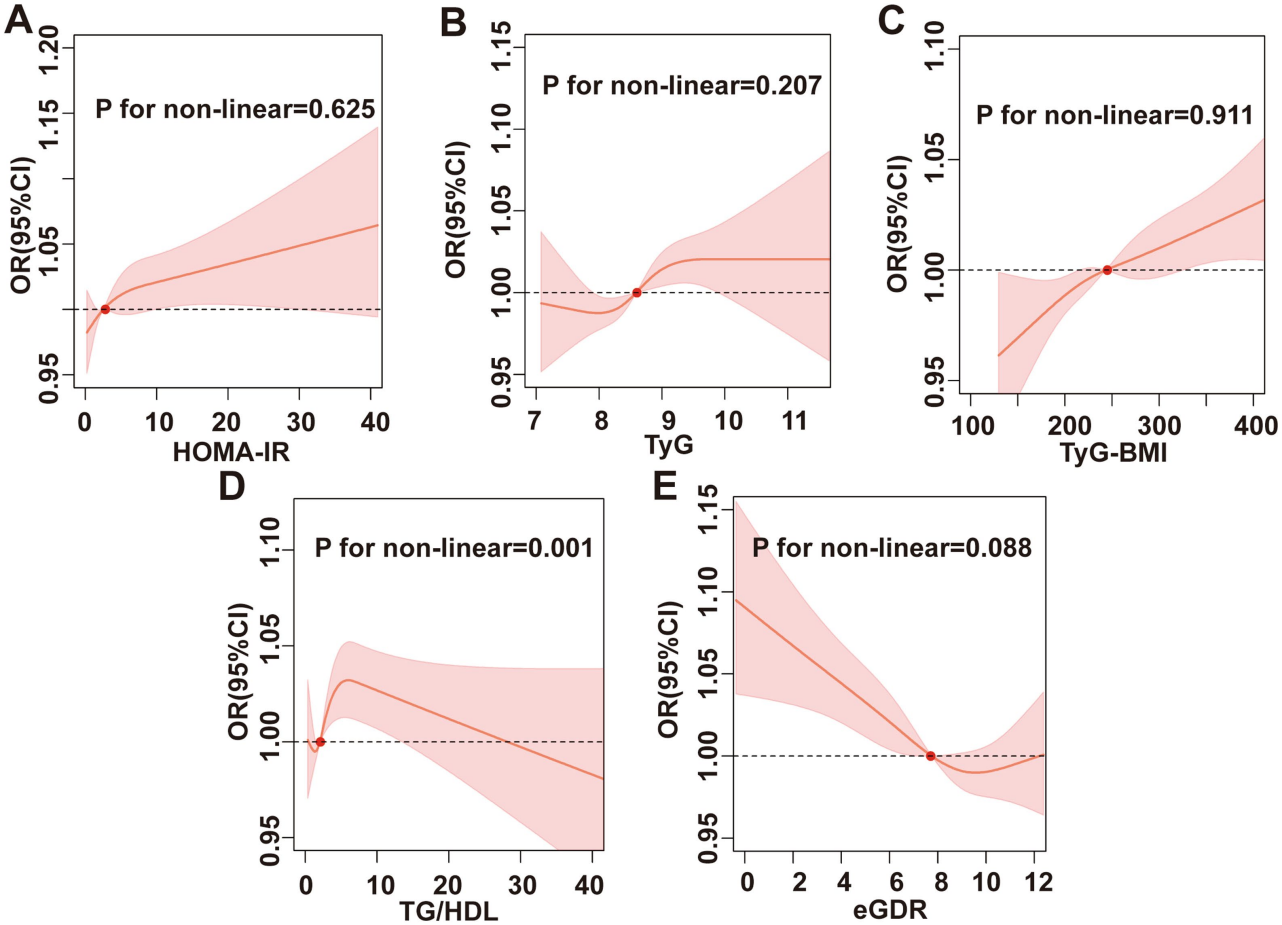
RCS analysis. Association between **(A)** HOMA-IR, **(B)** TyG index, **(C)** TyG-BMI, **(D)** TG/HDL, **(E)** eGDR, and chronic diarrhea.

The RCS results are illustrated in Figure 3. Except for TG/HDL, which exhibited a non-linear association with chronic diarrhea (*P* for non-linear = 0.001), TyG, eGDR, HOMA-IR, and TyG-BMI all demonstrated linear relationships with chronic diarrhea (*P* for non-linear > 0.05). Further threshold effect analysis revealed that each 1-unit increase in TG/HDL-C below the threshold of 7.33 elevated diarrhea risk by 11% (95% CI: 1.05–1.17) (Table 6).

**Table 6:** Threshold effect of IR surrogate indices.

Model	Adjusted OR (95%CI)	*P*-value
TG/HDL		
Logistic regression model	1.58 (0.19, 13.5)	0.700
Segmented regression model		
Inflection point		
<7.333	1.11 (1.05,1.17)	<0.001
>7.333	0.95 (0.89,1.00)	0.094
Log-likelihood ratio		

### 3.5 ROC curve analysis

**Figure 4:**
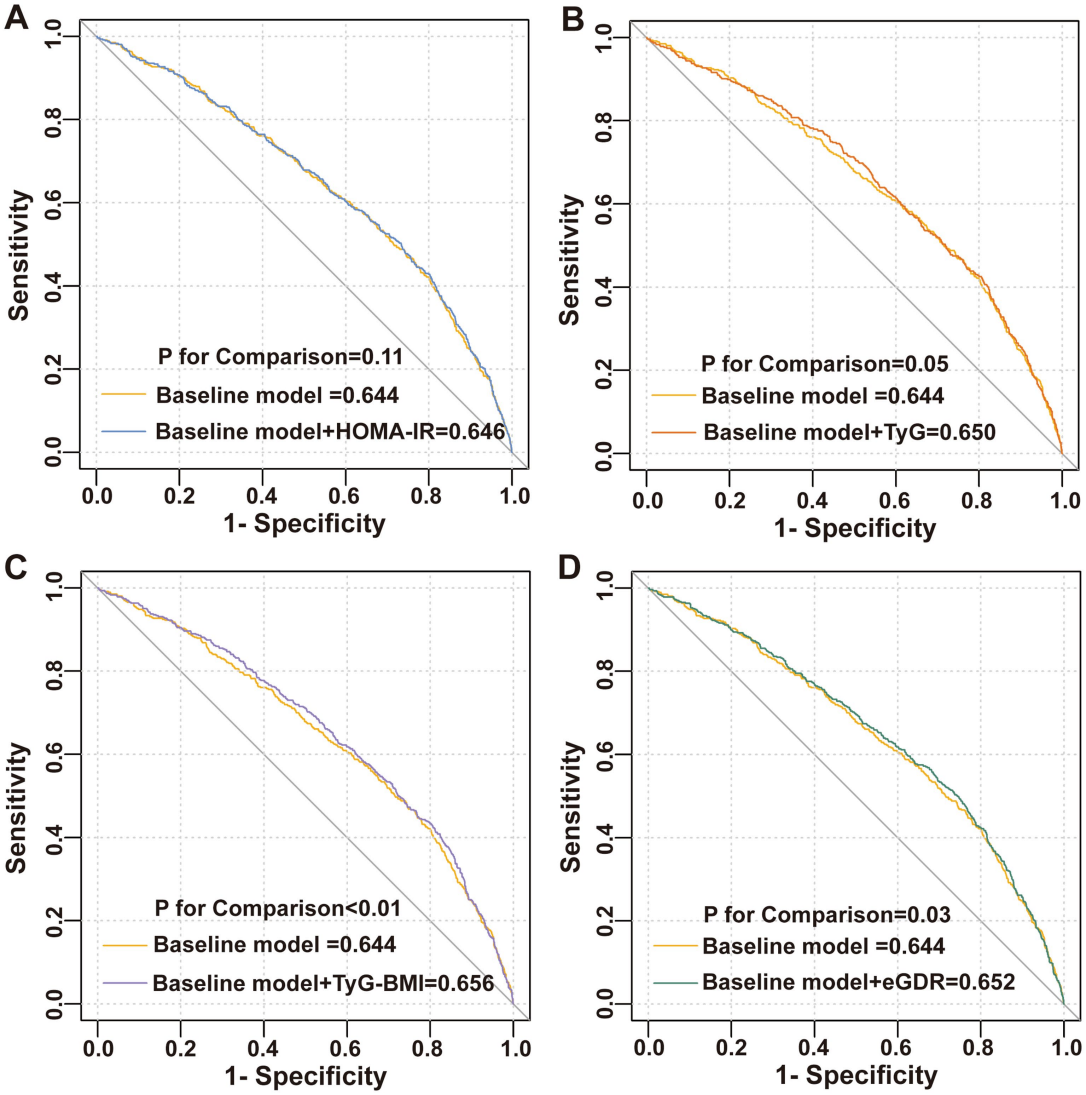
ROC curve. **(A)** HOMA-IR, **(B)** TyG index, **(C)** TyG-BMI, and **(D)** eGDR.

This study incorporated covariates from logistic Model 3 to establish a baseline risk model, aiming to evaluate the improvement in the discriminative ability of the model for diarrhea by adding IR surrogate indices. The results are illustrated in Figure 4. Although the inclusion of five IR surrogate indices enhanced the discriminative ability of the model for diarrhea, only the addition of TyG-BMI (AUC: 0.656 vs. 0.644, *p* < 0.01) and eGDR (AUC: 0.652 vs. 0.644, *p* = 0.03) demonstrated statistically significant improvements.

## 4 Discussion

This study comprehensively investigated the associations between five IR surrogate indices and chronic diarrhea and chronic constipation among U.S. adults. The results revealed that HOMA-IR, TyG, TyG-BMI, TG/HDL, and eGDR were significantly associated with chronic diarrhea in adults, and these associations were independent of diabetes, depressive symptoms, and PA levels. Furthermore, TyG-BMI and eGDR significantly improved the discriminative ability of the baseline model for chronic diarrhea. However, no evidence was found to support an association between these five IR surrogate indices and chronic constipation. These findings highlight the potential of IR surrogate indices as early predictive markers for chronic diarrhea, providing a novel theoretical foundation for the prevention and treatment of this condition.

IR is defined by diminished cellular sensitivity to insulin, leading to impaired glucose uptake, dysregulated hepatic glucose production, and altered lipid metabolism (31). Research indicates that IR surrogate indices hold significant clinical value in primary healthcare and have the potential for widespread application (32). However, studies investigating the relationship between IR surrogate indices and intestinal health remain limited. Existing evidence suggests that intestinal health is closely associated with factors such as gut microbiota, dietary habits, infections, and psychological states (33). Specifically, gut microbiota dysbiosis can compromise the mucosal immune barrier, leading to inflammatory responses, oxidative stress, and IR-related pathological processes (34). Animal studies further demonstrate that probiotics and *Lactobacillus plantarum* can modulate gut microbiota composition, improve glucose and lipid metabolism, and enhance insulin sensitivity (35). Clinical research has also shown that individuals with metabolic disorders, including diabetes (36), non-alcoholic fatty liver disease (37), metabolic syndrome (6), and obesity (13), are more susceptible to intestinal dysfunction. These findings suggest that IR may play a significant role in the development of abnormal intestinal health. Notably, a recent study reported a positive correlation between the TyG index and chronic diarrhea but found no significant association with chronic constipation (38). Building on this, our study further explored the relationship between multiple IR surrogate indices and intestinal health. The results revealed that several IR surrogate indices were significantly associated with chronic diarrhea but not with chronic constipation, consistent with previous findings. Currently, the relationship between IR and chronic constipation remains controversial, with no conclusive evidence supporting a direct link. This may be due to the study population not reaching the critical threshold for related risks. Future prospective studies are needed to clarify the causal relationship between IR and chronic constipation.

This study utilized RCS to elucidate the complex associations between IR surrogate indices and chronic diarrhea risk. Although multivariate logistic regression showed no significant correlation between TG/HDL ratio and chronic diarrhea, RCS analysis revealed a non-linear association, and threshold effect analysis further quantified this relationship: Each 1-unit increase in TG/HDL below 7.33 elevated chronic diarrhea risk by 11%. HOMA-IR, TyG, TyG-BMI, and eGDR exhibited linear dose–response relationships. Integrating IR surrogate indices into primary care practices can improve the accuracy of diagnoses for chronic diarrhea, thus promoting the creation of more tailored and effective management approaches. Specifically, populations showing heightened levels of IR surrogate indices can be selected for focused strategies to enhance intestinal health, which may involve more stringent monitoring, alterations in lifestyle, and strong pharmacological treatments to reduce their risk of chronic diarrhea. The intricacy of dietary questionnaires, along with the financial implications and discomfort linked to testing intestinal flora, presents considerable obstacles to their widespread use in primary care settings. In contrast, IR surrogate indices are easily accessible via standard blood tests and physical assessments, rendering them especially advantageous for primary care contexts.

This study demonstrated that only TyG-BMI and eGDR improved the discriminative ability of the baseline risk model for chronic diarrhea. Compared to HOMA-IR and TyG, the TyG-BMI and eGDR also incorporate BMI, effectively capturing the abnormal state of visceral fat accumulation in the body. Furthermore, individuals with diabetes and obesity are at a higher risk of experiencing chronic diarrhea (39, 40). Notably, pro-inflammatory cytokines released by visceral adipose tissue have been shown to impair intestinal barrier function and exacerbate chronic diarrhea (41, 42). Meanwhile, eGDR dynamically assesses insulin sensitivity and peripheral glucose disposal efficiency, offering a more comprehensive reflection of metabolic disturbances on intestinal energy metabolism and immune microenvironment (43). Although multivariate logistic regression revealed significant correlations between IR surrogate indices and chronic diarrhea, the discriminative capability of the ROC model was still limited, as indicated by an AUC below 0.75. Notably, previous studies on chronic diarrhea have also reported suboptimal AUC values. For instance, Yinda et al. found that the body roundness index (BRI) achieved an AUC of 0.606 for discriminating chronic diarrhea, outperforming both BMI (AUC: 0.569) and waist circumference (AUC: 0.572) (44). Due to data constraints, the current model did not incorporate gut microbiota-derived metabolites and dietary profiles. Future prospective studies should develop multidimensional predictive models by integrating metabolomic, microbiomic, and immunomic biomarkers to systematically unravel the IR-gut microenvironment interaction network, thereby optimizing risk stratification efficacy.

The precise pathological mechanisms underlying the relationship between IR surrogate indices and intestinal health remain incompletely understood but likely involve multiple interconnected pathways. First, IR-driven dysregulation of glucose and lipid metabolism compromises energy supply to intestinal smooth muscle, impairing its contractile function. Elevated blood glucose and insulin levels alter the excitability of intestinal nerves and musculature, resulting in motility disturbances that may present as constipation or diarrhea (45, 46). Second, systemic low-grade inflammation associated with IR elevates pro-inflammatory cytokines, which degrade intestinal barrier integrity and disrupt enteric nervous system signaling, further exacerbating motility dysfunction (47, 48). Third, IR is closely linked to gut microbiota dysbiosis. Such dysbiosis reduces short-chain fatty acid production, weakens the intestinal barrier, and facilitates endotoxin translocation, aggravating IR and intestinal impairment (49, 50). Fourth, IR may dysregulate gut–brain axis communication, perturbing the secretion of neurotransmitters (serotonin) and hormones (Glucagon-like peptide-1), which modulates intestinal motility and secretory activity (50–52). Finally, IR-related oxidative stress damages intestinal epithelial cells and neurons, impairing motility and barrier function, possibly contributing to constipation or diarrhea (53, 54). These interwoven mechanisms form a complex pathophysiological network that collectively drives chronic diarrhea or constipation.

To the best of our understanding, this research represents the initial attempt to analyze the relationships among IR surrogate indices and intestinal health. The robust quality control protocols and sophisticated sampling design implemented by NHANES facilitated the evaluation of correlations across various adult populations within the United States. In addition, the utilization of multivariate weighted logistic regression along with RCS analysis markedly improved the strength and dependability of our results. Notably, this investigation considered several confounding factors, such as socioeconomic status, social aspects, depression, PA levels, diabetes, and BMI, indicating that the findings of our study can be applied broadly.

While the results of this research provide novel insights into the early monitoring of chronic diarrhea and chronic constipation, several limitations warrant consideration. First, the cross-sectional design of the NHANES dataset restricts the ability to establish causal relationships between IR surrogate indices and intestinal health. Future studies should adopt a prospective design to validate these causal links. Second, the temporal scope of the data is confined to NHANES surveys conducted from 2005 to 2010, which limits external validation. While this study employs rigorous analytical methods, excluding residual confounding factors remains challenging. For instance, the gut microbiota, diet composition components, and medication usage may also influence the risk of diarrhea and constipation. In addition, the study population predominantly originates from the United States, which may restrict the external validity of the findings. Variations in dietary habits and lifestyles across different countries and regions could impact the applicability of the results to other populations. Therefore, further verification of the research findings in diverse geographical and cultural contexts is necessary to ascertain their universal applicability. Finally, due to the constraints of the NHANES dataset, more detailed clinical information required to apply the Rome IV criteria is not available; our assessment of intestinal health status relies solely on BSFS. Identifying chronic diarrhea and constipation through self-reported questionnaires may result in the potential misclassification. The limited scope of the intestinal health questionnaire hinders the collection of comprehensive data regarding various aspects of intestinal health, such as stool frequency and consistency, biochemical and/or microbial status, and duration and medication history for diarrhea. We agree that future research should incorporate both stool form and duration of symptoms to more accurately classify diarrhea subtypes.

## 5 Conclusion

This study identified significant associations between five IR surrogate indices and an elevated risk of chronic diarrhea. These findings suggest that IR surrogate markers hold promise as cost-effective, simple, and accessible early predictors for chronic diarrhea in high-risk populations.

## Data Availability

The original contributions presented in the study are included in the article/Supplementary material, further inquiries can be directed to the corresponding author.
