# Supplementary material for: Evaluating the role of insulin resistance in chronic intestinal health issues: NHANES study findings
Source: Front Nutr. 2025 May 26;12:1602922. doi: 10.3389/fnut.2025.1602922 (PMC12146163; doi:10.3389/fnut.2025.1602922)
Supplement: Supplementary file 1 [file Table_1.docx]

Supplementary Table1. Missing of variables

| Variables | Missing | Missing proportion |
| --- | --- | --- |
| Education | 6 | 0.09783140% |
| PIR | 435 | 7.09277678% |
| Smoke | 4 | 0.06522094% |
| Alcohol | 1 | 0.01630523% |
| BMI | 14 | 0.22827328% |
| ALT | 20 | 0.32610468% |
| AST | 20 | 0.32610468% |
| LDL | 144 | 2.34795369% |
| PA | 3 | 0.04891570% |
| Depressive symptom | 21 | 0.34240991% |
